# *NBEA* gene variant in a child with developmental disorder and epilepsy: a case report

**DOI:** 10.3389/fnins.2025.1662363

**Published:** 2025-10-03

**Authors:** Xiaoli Huang, Wen-Lin Wu, Jingjing Song, Yang Tian, Yilan Zhou, Shitao Wei, Bin Yu, Luoxiao Qin, Sida Yang

**Affiliations:** ^1^Department of Neurology, Liuzhou Hospital, Guangzhou Women and Children’s Medical Center, Liuzhou, China; ^2^Department of Neurology, Guangzhou Women and Children’s Medical Center, Guangzhou Medical University, Guangzhou, China

**Keywords:** *NBEA* gene, neurodevelopmental disorder, epilepsy, febrile sensitivity, whole-exome sequencing

## Abstract

The *NBEA* gene encodes Neurobeachin, a brain-specific kinase-anchoring protein that plays a critical role in vesicle trafficking and synaptic regulation. Pathogenic variants in *NBEA* are definitively associated with neurodevelopmental disorders accompanied by epilepsy, including intellectual disability, autism spectrum disorder, and myoclonic-astatic epilepsy–like phenotypes. Most reported disease-causing variants are *de novo* loss-of-function mutations, and although genotype–phenotype correlations remain limited, early-onset generalized seizures are frequently observed. Here, we describe a Chinese child presenting with global developmental delay and recurrent seizures with febrile sensitivity. Brain magnetic resonance imaging revealed no structural abnormalities, while electroencephalography showed epileptiform abnormalities. Genetic analysis identified a *de novo* nonsense variant in the *NBEA* gene: c.4715C > A [p.(Ser1572Ter)]. According to the American College of Medical Genetics and Genomics guidelines, the variant was classified as pathogenic. *NBEA* mutations are associated with neurodevelopmental disorders with or without early-onset epilepsy. Although no additional pathogenic variants were identified in the exome, the influence of other undetected genetic or epigenetic modifiers on the observed phenotype cannot be excluded. This case therefore refines the phenotypic spectrum of *NBEA*-related disorders, emphasizing that the c.4715C > A [p.(Ser1572Ter)] variant may be associated with developmental impairment and epilepsy with possible febrile sensitivity.

## 1 Introduction

The *NBEA* gene encodes Neurobeachin, a brain-specific kinase-anchoring protein involved in vesicle trafficking and the regulation of synaptic structure and function (Wang et al., 2000). Neurobeachin is predominantly localized to vesicular structures at the trans-Golgi network and within neuronal dendrites, where it facilitates the targeted trafficking of postsynaptic proteins (Nair et al., 2013; Niesmann et al., 2011). According to OMIM (OMIM #619157; updated 2023), *NBEA* is a definitively established disease-associated gene, linked to neurodevelopmental disorders with epilepsy, including autism spectrum disorder, intellectual disability, and various epilepsy phenotypes. Recent large-scale sequencing studies have identified *de novo NBEA* variants—primarily loss-of-function (LoF) mutations—in patients with NDDs, supporting its role in early-onset generalized epilepsy with phenotypes overlapping myoclonic-astatic epilepsy (MAE) (Jacobsen et al., 2015; Mulhern et al., 2018; Niesmann et al., 2011). The identical variant described in our case has been previously reported by Mulhern et al. (2018), in association with developmental delay and epilepsy, thereby limiting claims of allelic novelty while allowing for further refinement of the phenotype.

Approximately two-thirds of affected individuals present with epilepsy within the first 4 years of life, most commonly generalized seizures, including myoclonic, atonic, and myoclonic-atonic types. MAE-like presentations, often accompanied by developmental regression and speech delay, have been increasingly recognized in individuals with LoF variants in *NBEA*. However, genotype–phenotype correlations remain limited, and the full clinical spectrum associated with *NBEA* mutations is still being delineated (Mulhern et al., 2018).

Here, we report a Chinese pediatric case diagnosed with *NBEA*-related epilepsy at our center. Notably, the initial seizure occurred in the setting of fever, raising the possibility of febrile sensitivity associated with this gene—a phenotypic feature that has received little emphasis in the literature despite being noted in a small subset of individuals in previous cohort studies. Thus, the primary contribution of our study lies not in establishing *NBEA* as a disease gene or reporting a novel allele, but in expanding the phenotypic characterization—particularly regarding febrile sensitivity—in the context of an already documented pathogenic variant.

## 2 Manuscript formatting case description

A 14-months-old boy, the second child of non-consanguineous healthy parents, was born at term following an uncomplicated vaginal delivery. His birth history was unremarkable. Early developmental milestones were mildly delayed: he achieved head control at 4 months, independent sitting at 8 months, rolling over at 11 months, and pulling to stand at 14 months. His older sister was healthy, and there was no known family history of epilepsy or other neurological disorders.

**FIGURE 1:**
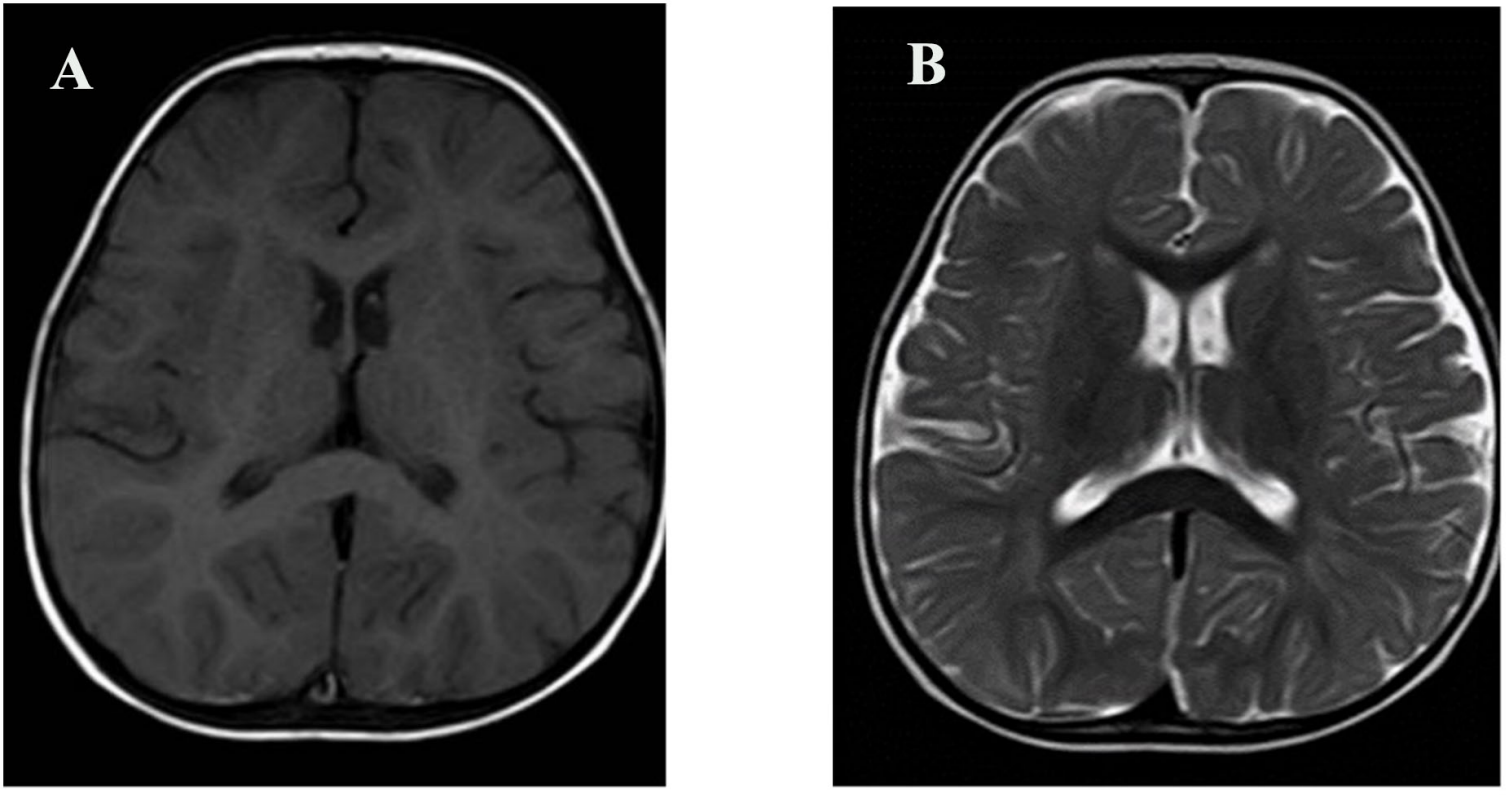
Brain MRI of the patient. **(A)** Axial T1-weighted image; **(B)** Axial T2-weighted image. No structural abnormalities were observed.

**FIGURE 2:**
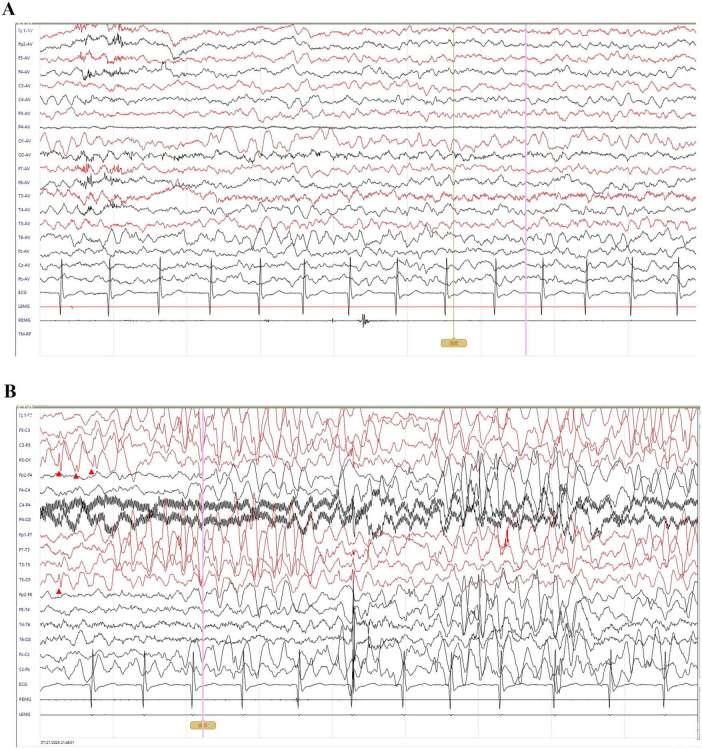
Interictal electroencephalography of the patient. All EEGs were recorded with a sensitivity of 10 μV/mm, timebase of 10 s/page, high-frequency filter at 70 Hz, and notch filter at 50 Hz; low-frequency filter was set to 0.5 Hz during wakefulness and 1 Hz during sleep. **(A)** Awake EEG shows a low-amplitude 6–7 Hz theta rhythm predominantly over the bilateral occipital regions, intermixed with low-amplitude fast activity. The rhythm is slightly irregular, indicating mildly impaired modulation. **(B)** Sleep EEG demonstrates bursts of ∼4 Hz medium-to-high amplitude theta activity diffusely across all channels, with occasional sharp–slow wave complexes predominantly in the left parietal and posterior temporal regions (red arrowheads).

At 11 months of age, he presented with his first seizure during a febrile illness, with a body temperature of 38°C–39°C. During this episode, seizures consistently occurred when the body temperature exceeded 38°C, with a seizure latency of within 1 h from the time the temperature rose above this threshold. The seizure was generalized tonic-clonic in type and lasted 1–3 min. During the same febrile episode, he experienced five to six similar seizures within a short period. He was admitted to our department on 4 November 2021, for evaluation. Brain magnetic resonance imaging was unremarkable (Figure 1). Interictal electroencephalography demonstrated mildly slowed occipital background activity (6–7 Hz theta rhythm with intermixed low-amplitude fast activity) during wakefulness, and sparse sleep spindles with occasional left parietal–posterior temporal sharp–slow wave complexes during sleep (Figure 2). Routine laboratory tests, metabolic screening, cardiac ultrasound, and electrocardiogram were all within normal limits. Developmental assessment using the Gesell scale at 11 months of age revealed a global developmental quotient of 73, with scores of 60 for gross motor (able to roll over and sit without support, able to pull to stand with assistance, but unable to stand independently), 53 for fine motor (immature pincer grasp, limited ability to transfer small objects between hands), 70 for cognitive (recognizes familiar people, responds to name, engages briefly with simple toys), 73 for language (produces several babbling sounds and 2–3 single meaningful words, without combining words), and 70 for social skills (maintains eye contact, smiles responsively, responds to social cues from caregivers, but shows limited interaction with peers). Complex febrile seizures were suspected, and the patient improved with symptomatic treatment and was discharged after three days.

On 30 November 2021, he was re-admitted for recurrent febrile seizures—six episodes within a single day—again associated with a fever reaching 39°C. On this occasion, each seizure occurred within 1 h after the body temperature exceeded 38°C, with the maximum recorded temperature before seizure onset being 38.8°C–39.0°C. At that time, no further etiology was identified, and complex febrile seizures remained the working diagnosis.

**FIGURE 3:**
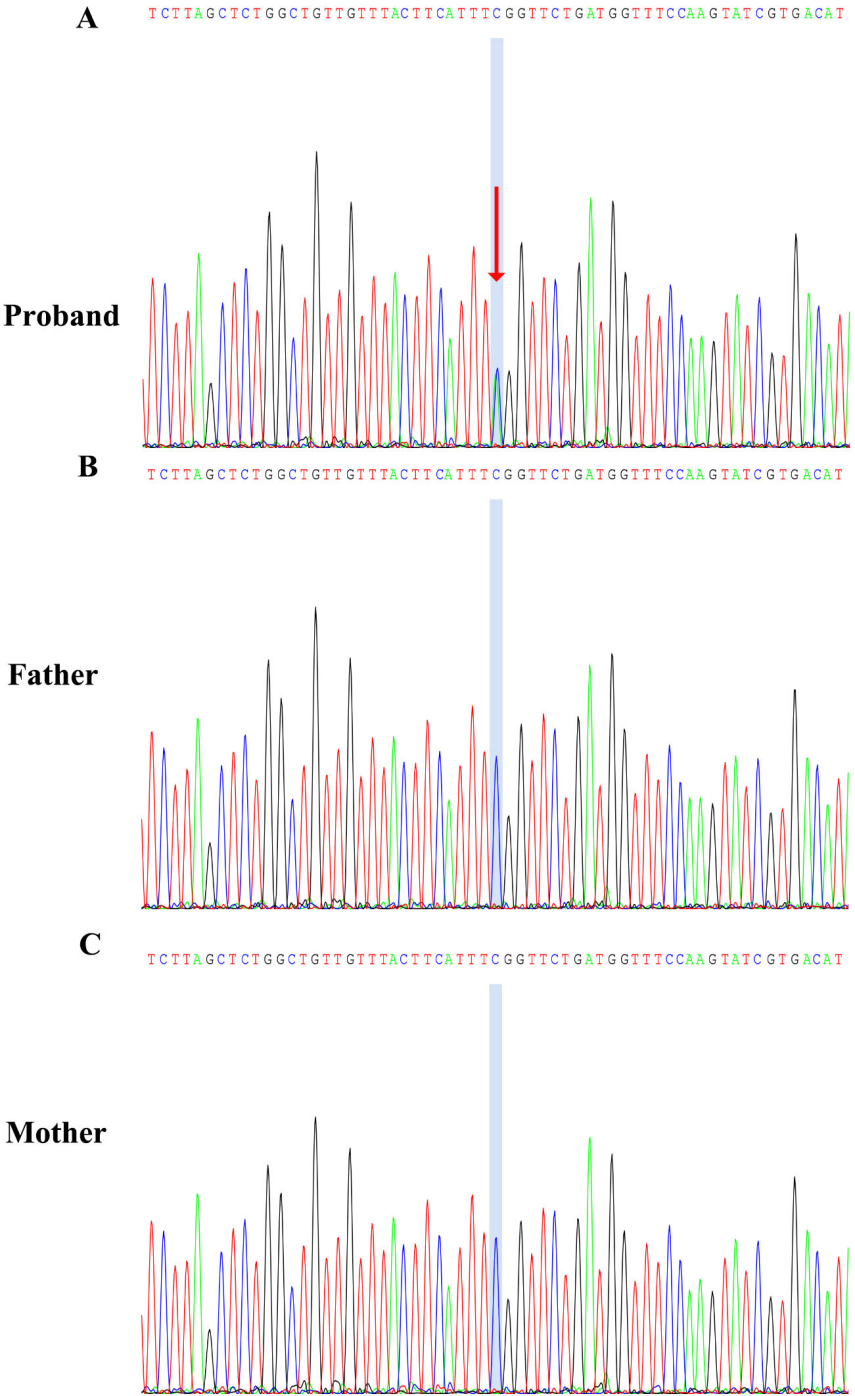
Sanger sequencing confirmation of the *NBEA* c.4715C > A [p.(Ser1572Ter)] variant. **(A)** Proband showing a heterozygous C > A substitution, indicated by overlapping peaks at the variant site (blue shading and red arrow). **(B)** Father carrying the wild-type sequence at the same locus. **(C)** Mother also carrying the wild-type sequence.

**FIGURE 4:**
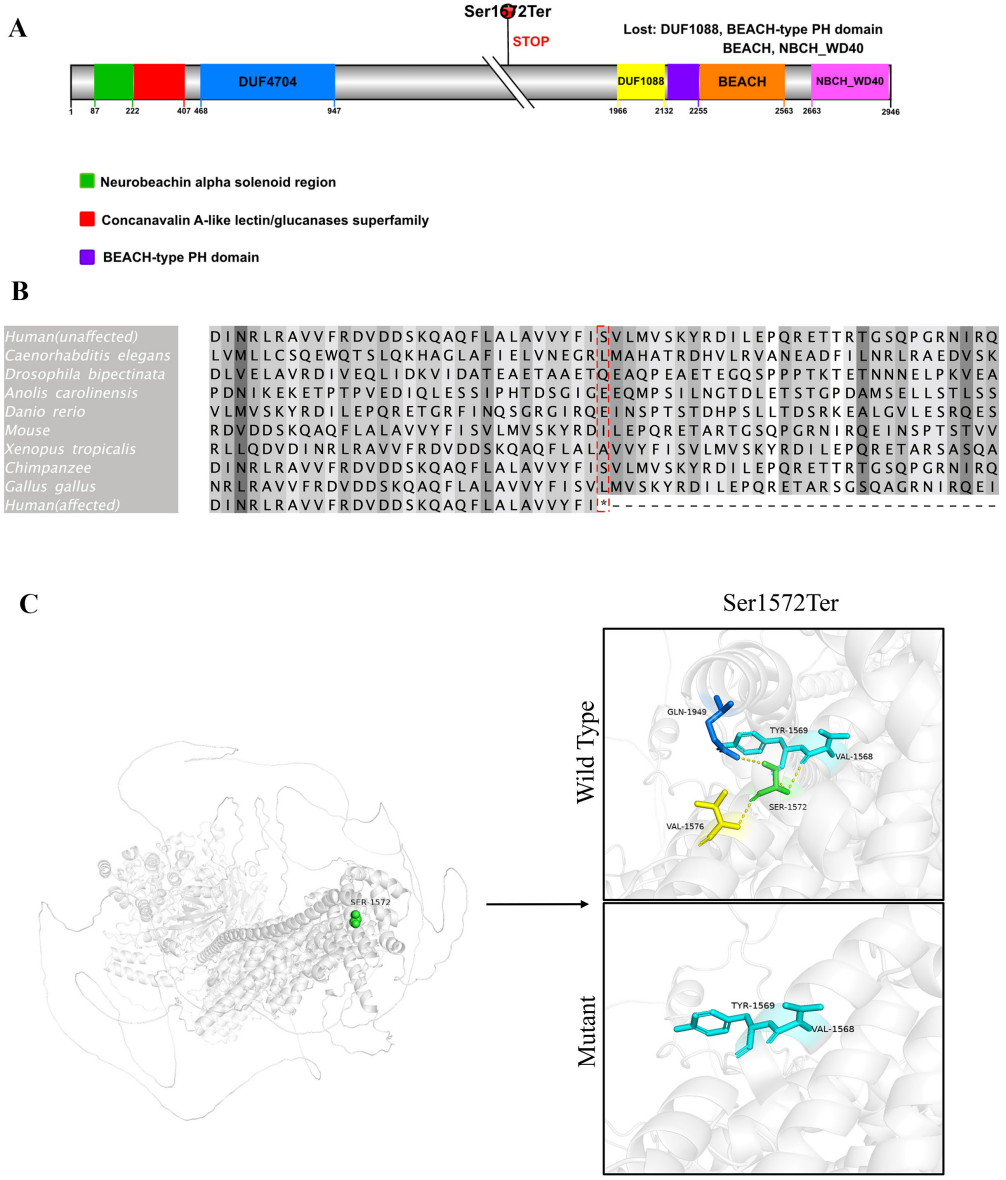
Domain architecture, evolutionary conservation, and structural impact of the p.(Ser1572Ter) variant in *NBEA*. **(A)** Schematic representation of the human Neurobeachin (NBEA) protein (UniProt ID: Q8NFP9) showing the location of the c.4715C > A [p.(Ser1572Ter)- non-sense variant. The mutation is indicated by a red circle above the schematic and results in premature termination upstream of the C-terminal domains. Colored boxes represent annotated domains: green, neurobeachin alpha helical region; red, concanavalin A-like lectin/glucanases superfamily; purple, BEACH-type PH domain. Numbers below indicate amino acid positions. **(B)** Multiple sequence alignment of the protein region containing the mutation. Alignment was performed using Clustal Omega in Jalview (version 2.11.4.1). Species and reference sequence accession numbers are as follows: *H. sapiens* (NP_056493.3, unaffected; NP_056493.3, affected), Pan troglodytes (XP_016780658.1), *M. musculus* (XP_036019004.1), *G. gallus* (XP_025008670.1), *Anolis carolinensis* (XP_016848095.1), *Xenopus tropicalis* (XP_012813375.1), *D. rerio* (XP_009290096.1), *C. elegans* (NP_001364559.1), *D. bipectinata* (XP_017102372.1). The red box highlights the mutation site in the affected human sequence; shading intensity indicates residue conservation across species. **(C)** Structural comparison of wild-type and mutant *NBEA* protein at residue 1,572. In the wild-type model (upper panel), Ser1572 (green sticks) forms hydrogen bonds (yellow dashed lines) with surrounding residues Tyr1569, Val1568, Val1576, and Gln1949, stabilizing the local structure. In the mutant model (lower panel), the p.(Ser1572Ter) non-sense mutation leads to premature termination, resulting in loss of Ser1572 and all subsequent residues, abolishing these interactions. Protein backbone is shown in gray ribbon representation, with interacting residues colored according to atom type. Three-dimensional structures were predicted using AlphaFold v3.0 and visualized with PyMOL Molecular Graphics System, Version 2.1 (Schrödinger, LLC).

Three months later, the patient began experiencing afebrile seizures with similar semiology. Given the evolution to unprovoked seizures, genetic testing was pursued. Whole-exome sequencing revealed a *de novo* heterozygous non-sense variant in the *NBEA* gene (NM_001385012.1): c.4715C > A, resulting in a premature stop codon [p.(Ser1572Ter)] (Figure 3). Sanger sequencing confirmed the variant was absent in both parents. Whole-exome sequencing also revealed three additional variants. A heterozygous missense variant in *BRPF1* {NM_001003694.2:c.2339T > A [p.(Leu780Gln)]} was classified as of uncertain significance and has been reported in association with intellectual developmental disorder with dysmorphic facies and ptosis (MIM:617333) in an autosomal dominant inheritance pattern. Two heterozygous frameshift variants were also identified: *ALDOB* {NM_000035.4:c.360_363del [p.(Asn120fsTer32)]}, previously reported as pathogenic for hereditary fructose intolerance (MIM:229600) with autosomal recessive inheritance; and *HBB* {NM_000518.5:c.126_129del [p.(Pro42fsTer19)]}, a known pathogenic variant for β-thalassemia (MIM:613985), also inherited in an autosomal recessive manner. These variants are unlikely to account for the patient’s neurological phenotype because they are either recessive or of uncertain significance. Importantly, none of them represent rare truncating variants in autosomal dominant genes or in genes with high probability of loss-of-function intolerance. In addition to the *de novo* NBEA variant and the three variants in genes with established disease associations (BRPF1, ALDOB, and HBB), whole-exome sequencing did not reveal any rare candidate variants in genes without known gene–disease association. Therefore, the *NBEA* c.4715C > A [p.(Ser1572Ter)] variant remains the most plausible molecular explanation for the clinical presentation. No pathogenic variants were identified in the mitochondrial genome. The variant is predicted to lead to non-sense-mediated mRNA decay (NMD), consistent with a loss-of-function mechanism. It was not found in population databases such as the 1,000 Genomes Project, gnomAD, or ClinVar. The same variant had been previously reported once in the literature as a *de novo* variant in a patient with neurodevelopmental delay. According to ACMG guidelines, it was classified as pathogenic. To further explore the potential functional consequences of this variant, we performed domain mapping, evolutionary conservation analysis, and structural modeling (Figure 4). To assess the location and potential impact of the c.4715C > A [p.(Ser1572Ter)] variant in *NBEA*, we first generated a schematic representation of the NBEA protein (Figure 4A). The variant is located between the DUF4704 and DUF1088 domains and introduces a premature stop codon at residue 1,572, resulting in the loss of several C-terminal conserved domains. Multiple sequence alignment showed that the affected residue is well-conserved across vertebrate and invertebrate species, including *Homo sapiens*, *Mus musculus*, *Danio rerio*, *Gallus gallus*, *Caenorhabditis elegans*, and *Drosophila bipectinata*. The position of the premature stop codon in the affected human sequence is highlighted in red, and shading intensity reflects the degree of residue conservation (Figure 4B). Three-dimensional structural modeling (Figure 4C) revealed that, in the wild-type protein, Ser1572 forms a network of hydrogen bonds with Tyr1569, Val1568, Val1576, and Gln1949, contributing to local structural stability. In the mutant model, premature truncation leads to the loss of Ser1572 and all downstream residues, abolishing these interactions. A diagnosis of epilepsy was established, and oral sodium valproate was initiated. The initial dose was 1.85 ml twice daily, which was gradually titrated to 2.5 ml twice daily. On 14 February 2022, a brief febrile focal aware seizure occurred, presenting with upward gaze and head nodding without secondary generalization. His valproate dose was subsequently increased to 4 ml twice daily. Following another febrile seizure in June 2023, the dose of valproate was increased to 5 ml twice daily (approximately 29 mg/kg/day). Since that adjustment, the patient has remained seizure-free for over 18 months.

## 3 Discussion

Neurobeachin is a neuron-specific, multidomain scaffolding protein with a molecular weight of approximately 327 kDa. It belongs to the BEACH (Beige and Chediak-Higashi) domain-containing protein family and plays a critical role in vesicle trafficking and synaptic function (Wang et al., 2000). In Wang et al. (2000) demonstrated that Neurobeachin predominantly localizes to the trans-Golgi network tubular-vesicular membranes in neurons through brefeldin A (BFA) diffusion assays. This recruitment is promoted by GTPγS and inhibited by BFA, indicating that NBEA participates in regulated vesicle dynamics (Wang et al., 2000). It is abundantly expressed in the brain and is essential for synaptic development in both the central nervous system and neuromuscular junctions (Nair et al., 2013). In zebrafish, postsynaptic Neurobeachin is required for the formation of electrical and chemical synapses and the maintenance of dendritic complexity (Niesmann et al., 2011). Knockout mouse models have shown that complete loss of *NBEA* results in perinatal lethality, further supporting its essential role in neurodevelopment (Odent et al., 2021).

According to OMIM (OMIM #619157; updated 2023), *NBEA* is a definitively established disease-associated gene linked to neurodevelopmental disorders with epilepsy. *NBEA* was initially considered a candidate gene for autism based on expression patterns and genomic rearrangements in affected individuals. However, recent studies have expanded its known phenotypic spectrum to include NDDs with or without early-onset generalized epilepsy (Boulin et al., 2021; Jacobsen et al., 2015; Miller et al., 2015). The disorder follows an autosomal dominant inheritance pattern, and pathogenic variants—most commonly non-sense, frameshift, or splice-site mutations—are predicted to result in LoF, primarily through non-sense-mediated mRNA decay (Jacobsen et al., 2015).

Language delay is a common clinical feature, and approximately half of individuals with *NBEA* variants develop epilepsy. Most affected individuals experience global developmental delay or intellectual disability, along with speech delay and behavioral abnormalities (Jacobsen et al., 2015; Miller et al., 2015; Mulhern et al., 2018). Seizure onset typically occurs within the first year of life. Myoclonic and myoclonic-atonic seizures are among the most common seizure types, frequently resembling MAE (Mulhern et al., 2018).

In the study by Mulhern et al. (2018), 24 patients with *de novo NBEA* variants were characterized. All patients exhibited neurodevelopmental impairment, and approximately two-thirds had epilepsy. Notably, seizure types and severity varied by genotype (Mulhern et al., 2018). Myoclonic and atonic seizures were more common in individuals with LoF variants, whereas missense variants were associated with a broader and often milder phenotype. Febrile sensitivity was reported in two individuals: one with a splice-site variant (c.7707+2T > C) and another with a missense variant [c.8401G > A (p.Glu2801Lys)], both experiencing seizures during febrile episodes. Importantly, the identical non-sense variant identified in our patient {c.4715C > A [p.(Ser1572Ter)]} was also described by Mulhern et al. (2018), in a patient who exhibited paroxysmal spells without confirmed seizures, indicating that this allele is not novel. Our patient also harbors the c.4715C > A [p.(Ser1572Ter)] *de novo* non-sense variant but presented with recurrent febrile seizures followed by afebrile generalized tonic-clonic seizures, along with global developmental delay. This expands the phenotypic characterization of this known pathogenic variant, highlighting febrile sensitivity as a potential but underrecognized component of the *NBEA*-associated epilepsy spectrum. This contrast underscores the clinical heterogeneity of *NBEA*-associated disorders—even among individuals carrying the same genotype, phenotypic expression can range from no confirmed seizures to recurrent generalized epilepsy with febrile sensitivity. Conversely, individuals with different genotypes may exhibit similar seizure types, including febrile-onset epilepsy, reflecting significant genotype–phenotype variability.

The clinical heterogeneity observed in *NBEA*-associated disorders, even among individuals harboring identical variants, is consistent with broader neurodevelopmental epilepsy genetics, where modifier genes, epigenetic regulation, and environmental factors can profoundly shape phenotypic expression. Similar patterns have been reported for *SCN1A* and *KCNQ2*, in which the same pathogenic variant may present as severe epileptic encephalopathy or self-limited epilepsy depending on the genetic and cellular context (Brunklaus et al., 2014; Lersch et al., 2023). This variability may also explain the presence or absence of febrile sensitivity in individuals with the same *NBEA* allele.

At the mechanistic level, NBEA is a multidomain scaffolding protein essential for postsynaptic receptor trafficking, synaptic organization, and compartment-specific protein localization (Farzana et al., 2016; Lützenkirchen et al., 2024; Martin et al., 2023; Miller et al., 2015; Tuand et al., 2016; Volders et al., 2012; Wang et al., 2000). The PH–BEACH–WD40 module mediates AMPA/NMDA receptor targeting, while the Armadillo domain regulates actin-dependent filopodia formation, and WD40/DUF1088 domains facilitate SAP102 binding and ion channel localization (Farzana et al., 2016; Lützenkirchen et al., 2024; Volders et al., 2012). Beyond chemical synapses, NBEA interacts with ZO1 to maintain dendrite-specific localisation of electrical synapse proteins (Martin et al., 2023; Miller et al., 2015). Additionally, the A-kinase anchoring protein domain spatially confines protein kinase A activity, and *NBEA* haploinsufficiency can produce bidirectional dysregulation of protein kinase A -dependent phosphorylation, altering actin cytoskeletal remodeling and receptor trafficking (Tuand et al., 2016; Wang et al., 2000). Such effects are potentially temperature-sensitive, providing a plausible link between NBEA dysfunction and febrile-onset seizures in susceptible individuals. These converging roles across synapse types, together with functions in transcriptional control, provide a coherent molecular basis for the broad phenotypic spectrum—including epilepsy, autism spectrum disorder features, and febrile sensitivity—observed in NBEA-related neurodevelopmental disorders. Although whole-exome sequencing did not reveal any additional pathogenic or likely pathogenic variants in our patient, we cannot exclude the contribution of other undetected genetic or epigenetic modifiers to the observed phenotype.

Our analysis shows that the p.(Ser1572Ter) variant lies within a well-conserved region of NBEA and introduces a premature stop codon upstream of several essential C-terminal domains, suggesting a potentially deleterious effect on protein function. Conservation analysis indicates that this residue is preserved across diverse vertebrate and invertebrate species, supporting its functional relevance. Structural modeling revealed that Ser1572 participates in a hydrogen-bond network stabilizing the local structure, and its loss abolishes these interactions. Moreover, the truncation removes four key domains—DUF1088, BEACH-type PH domain, BEACH, and NBCH_WD40—which are implicated in vesicle trafficking, membrane dynamics, and signal transduction. The absence of these domains is likely to severely compromise NBEA’s role in synaptic vesicle release and neuronal plasticity. Previous reports identified two pathogenic variants in the C-terminal WD40 domain associated with focal and febrile-sensitive seizures, and a DUF1088 variant that disrupted neuronal potassium channel trafficking in a *C. elegans* model (Boulin et al., 2021; Lauks et al., 2012; Repetto et al., 2018). Both domains mediate SAP102 binding within the PH–BEACH module and regulate AMPA/NMDA receptor trafficking (Lauks et al., 2012). Variants in this DUF1088–PH–BEACH–WD40 region may impair ion channel and receptor targeting, lowering seizure threshold. In our patient, the p.(Ser1572Ter) truncation upstream of these domains is expected to abolish these interactions, potentially facilitating temperature-sensitive seizure onset via disrupted receptor trafficking under febrile stress. Given these mechanistic insights and the emerging association between *NBEA* variants and febrile sensitivity, we propose that *NBEA* could be considered for inclusion in targeted genetic screening panels for children with unexplained febrile-sensitive epilepsy, particularly when established panel genes yield negative results, to facilitate early diagnosis, counseling, and anticipatory management.

Treatment response in *NBEA*-related epilepsy has been variable. According to the cohort reported by Mulhern et al., some patients achieved seizure remission between ages 3 and 19, while others remained refractory to treatment (Mulhern et al., 2018). Various antiseizure medications have been used with mixed results, including valproic acid, ethosuximide (often in combination with valproate), levetiracetam, lamotrigine, benzodiazepines, and dietary therapies (Mulhern et al., 2018). In our case, sustained seizure control was achieved after dose adjustment of valproic acid, suggesting that some patients may respond favorably to conventional therapy, although treatment must be individualized. Given the established disease association of *NBEA* and prior documentation of the same variant, the primary contribution of this report lies in refining genotype–phenotype correlations—particularly in relation to febrile sensitivity—and adding clinically relevant detail to the management of *NBEA*-related epilepsy.

A limitation of our study is that no additional pathogenic variants were identified in the exome that could account for the observed phenotypic differences. Therefore, the possibility of other undetected genetic or epigenetic modifiers cannot be excluded.

## 4 Conclusion

The c.4715C > A [p.(Ser1572Ter)] variant in the *NBEA* gene expands the mutational spectrum of *NBEA*-related disorders. This pathogenic variant is associated with neurodevelopmental impairment accompanied by epilepsy, and in this case, the seizures demonstrated febrile sensitivity.

## Data Availability

The original contributions presented in the study are included in the article. Further inquiries can be directed to the corresponding author.
